# Jinlida Granules Improve Dysfunction of Hypothalamic-Pituitary-Thyroid Axis in Diabetic Rats Induced by STZ

**DOI:** 10.1155/2018/4764030

**Published:** 2018-06-06

**Authors:** Chaoqun Wang, Xianliang Dai, Danfeng Zhang, Zhimin Liu, Qin Huang

**Affiliations:** ^1^Department of Endocrinology, Changhai Hospital, Second Military Medical University, Shanghai 200003, China; ^2^Department of Endocrinology, Changzheng Hospital, Second Military Medical University, Shanghai 200003, China; ^3^Department of Cardiology, Changzheng Hospital, Second Military Medical University, Shanghai 200003, China; ^4^Department of Neurosurgery, Changzheng Hospital, Second Military Medical University, Shanghai 200003, China

## Abstract

**Objective:**

We aim to explore the effects and mechanisms of Jinlida granules on the dysfunction of hypothalamic-pituitary-thyroid (HPT) axis in diabetic rats induced by streptozotocin.

**Methods:**

A total of 48 SD rats were randomized into normal control group (NC, *n* = 6) and diabetic group (*n* = 42). Rats in diabetic group were randomly divided into diabetes mellitus (DM) control group, low, medium, and high doses of Jinlida group (JL, JM, and JH), medium dose of Jinlida plus Tongxinluo group (JM + T), metformin group (Met), and Saxagliptin group (Sax) (*n* = 6 in each group). Diabetic rats were obtained by intraperitoneal injection of streptozotocin and sacrificed at 8 weeks to examine the function of HPT axis.

**Results:**

Levels of fasting blood glucose (*P* < 0.05), pI*κ*B, TNF*α* (*P* < 0.05), pNF-*κ*B, and IL-6 (*P* < 0.01) in liver tissue and TSHR mRNA expression (*P* < 0.01) in diabetic group were significantly increased, while levels of serum T3 and T4, thyroid hormone receptor (TR) mRNA and Dio1 mRNA in liver tissue, and sodium iodide symporter (NIS) mRNA in thyroid tissue in diabetic group were significantly decreased compared with those in NC group (*P* < 0.01). Among diabetic groups, level of fasting blood glucose in JH, JM + T and Met group was lower (*P* < 0.05) compared with DM group. However, levels of serum T3 and T4, TR mRNA in liver tissue, TSHR, and NIS mRNA in thyroid tissue in JH, JM + T, Met, and Sax group were significantly increased (*P* < 0.01) compared to DM group. In contrast, levels of Dio1 mRNA, pI-*κ*B in Met and JM + T groups, pNF-*κ*B in JH, JM + T, and Met group, and TNF*α* and IL-6 in JM, JH, JM + T, and Met group were significantly decreased (*P* < 0.05). HE staining showed reduced thyroid follicular epithelium and follicular area, as well as increased colloid area in DM group, indicating impaired synthesis, reabsorption, and secretory of TH in diabetes, which was significantly improved in JH, JM + T, and Met groups.

**Conclusion:**

HPT axis dysfunction in DM could be significantly improved by Jinlida granules. The mechanism might be associated with the anti-inflammatory effects involving NF-*κ*B pathway. Our findings suggested the potential benefit of Jinlida granules for patients with HPT axis dysfunction and DM, which was to be verified by more experimental and clinical studies.

## 1. Introduction

Thyroid diseases and diabetes mellitus (DM) represent two most common endocrine disorders, both of which involve multiple organs. The common predisposing genes are detected for DM and thyroid diseases by Genome-Wide Association Study [1]. Various clinical studies suggest a significantly increased incidence of thyroid dysfunction in patients with DM [2, 3], and one study indicates that the ratio of TT4, TT3, FT3, and TT3/rT3 in DM patients is significantly lower compared to normal group [4].

Clinically, thyroid dysfunction is closely related to insulin resistance [5]. Hyperthyroidism can increase glucose production, absorption, and utilization, leading to hyperinsulinemia, abnormal glucose tolerance, and peripheral insulin resistance [6]. The relationship between hypothyroidism and insulin resistance has been demonstrated in vitro and vivo [7–9]. Subclinical hypothyroidism has also been reported to be associated with insulin resistance [10–12]. A meta-analysis shows that subclinical hypothyroidism is associated with an increased risk of diabetic peripheral neuropathy, diabetic peripheral arterial disease, diabetic nephropathy, and diabetic retinopathy in patients with T2DM [13]. Therefore, diabetes can cause thyroid dysfunction, which, in turn, exacerbates DM and its complications.

Jinlida granule, a traditional Chinese medicine including 17 medicinal components (Table 1), is used for the treatment of T2DM. Lots of studies have shown that Jinlida granules could improve insulin resistance, promote insulin secretion, and reduce blood lipids [14–17]. It has been approved by the China Food and Drug Administration for the treatment of T2DM. However, it is yet unknown about the relationship between Jinlida granules and TH level in DM. In the present study, we aim to evaluate the effect of Jinlida granules on levels of hypothalamic-pituitary-thyroid (HPT) axis hormones, TH receptor, and thyroid stimulating hormone (TSH) receptor in diabetic rats induced by streptozotocin (STZ).

## 2. Materials and Methods

### 2.1. Animals

A total of 48 8-week-old male SD rats with an average weight of 200 g were purchased from Shanghai SLAC Laboratory Animal Co., Ltd. (Shanghai, China). Animals were housed in a climate-controlled space with a 12-hour light and dark cycle and provided with mouse chow and water ad libitum.

### 2.2. Induction of Diabetic Rats

After 1 week of acclimation, rats were randomly divided into the control (*n* = 6) and diabetic group (*n* = 42). Rats in the diabetic group were intraperitoneally injected with a single dose of 60 mg/kg (2.0 ml/kg) of STZ. On the third day after the injection, blood samples were taken to examine the blood glucose. Rats with blood glucose exceeding 16.7 mmol/L were deemed as diabetic. Rats in diabetic group were randomly divided into diabetic control group (no drug intervention group, DM), low, medium, and high doses of Jinlida group, medium dose of Jinlida plus Tongxinluo group (JM + T), metformin group (Met), and Saxagliptin group (Sax) (*n* = 6 in each group). Intragastric administration was performed at 16:00 p.m. every day for 8 weeks, and animals were treated with corresponding drugs dissolved in 0.5% Na-CMC. Animals in diabetic model group and blank control group were intragastrically administrated with 0.5% Na-CMC at the same dose. Volume of intragastric administration for each rat was about 1 ml per 100 g weight, and the used dose and method in each group were illustrated in Table 2. After 8 weeks of administration and 12-hour fasting, rats were sampled for blood glucose test and then anesthetized with sodium pentobarbital and sacrificed. Blood samples were harvested from the carotid artery. Thyroid, liver, hypothalamus, pituitary, and other tissues were extracted in minutes.

### 2.3. RNA Extraction, and Reverse Transcription-Quantitative Polymerase Chain Reaction (RT-qPCR)

Total RNA was extracted from the tissues using TRIzol reagent. The purity of obtained RNA was estimated using a NanoDrop 2000c spectrophotometer (Thermo Fisher Scientific, Inc., Wilmington, DE, USA), and RNA with an A260/A280 ratio of >1.8 was used for cDNA synthesis. First strand cDNA synthesis was performed using the First Strand cDNA Synthesis kit (TakaRa BIO Inc., Japan Cat. No. 6210A). qPCR was performed using SYBR Green PCR Master Mix iQ (Bio-Rad Laboratories, Inc. Singapore Cat. No. 170-8882AP) and CFX Connect Real-Time PCR System (Bio-Rad Laboratories, Inc., Hercules, CA, USA) according to the manufacturer's protocols. qPCR conditions were as follows: 10 min at 95°C, followed by two-step PCR at 95°C for 15 sec and 60°C for 1 min for 40 cycles with fluorescence monitoring at the end of each elongation step. Primers were obtained from Thermo Fisher Scientific, Inc. (Waltham, MA, USA) and listed in Table 3. Relative mRNA expression of target genes was calculated using the 2^−ΔΔCq^ method. Target sequences were normalized to GAPDH in multiplexed reactions performed in duplicate.

### 2.4. Determination of the Content of T3, T4, and TSH

The content of T3 and T4 was analyzed by Roche Cobas E602 automatic electrochemiluminescence analyzer according to the manufacturer's protocols. The content of TSH was detected by radioimmunoassay, which was processed automatically by gamma radiation immunoassay analyzer.

### 2.5. Hematoxylin and Eosin (H&E) Staining

Standard H&E staining was performed according to the manufacturer's protocol (Beijing Xinhualvyuan Science and Technology, Ltd., Beijing, China) and Olympus IX51 (Olympus Corporation, Japan) was used to view the staining.

### 2.6. Western Blot Analysis

Cells were lysed with modified RIPA lysis buffer (50 mM Tris-HCl pH 7.4, 150 mM NaCl, 1 mM EDTA, 1% Triton X-100, 1 mM NaF, 1 mM Na2VO4, 1 mM PMSF, and protease inhibitor cocktail). After incubation on ice and centrifugation at 12,000 rpm for 20 minutes, the supernatants were collected and protein concentration was measured by BCA (Bicinchoninic acid) method. Equal amounts of protein samples were mixed with 5x loading dye buffer and heated for 5 minutes at 95°C. Protein was resolved by sodium dodecyl sulfate polyacrylamide gel electrophoresis (SDS-PAGE) and then transferred to a nitrocellulose membrane by electroblotting. Membranes were incubated overnight at 4°C with anti-p-NF-KB, anti-P-IKB, anti-GAPDH (Cell Signaling Technology), anti-IL-6, anti-TNF-*α* (abcam), anti-Dio1, anti-NIS (ABclonal), and anti-*β*-actin (Cell Signaling Technology). Then different HRP-labeled secondary antibodies were diluted 1 : 1000 and incubated with the membrane at 37°C for 1 h, respectively. Signals were detected using the enhanced chemiluminescence procedure by LI-COR ODYSSEY.

### 2.7. Statistical Analysis

Data with normal distribution were expressed as mean ± standard deviation (SD) and compared using Student's *t*-test for differences between two groups, one-way analysis of variance for differences among multiple groups, followed by Fisher's least significant difference test. Results with skewed distribution were expressed as median and interquartile range (IQR) and tested with nonparametric test. *P* < 0.05 was considered as statistically significant. All statistical analyses were conducted using SPSS 19.0 (IBM SPSS, Armonk, NY, USA).

## 3. Results

### 3.1. Body Weight and Blood Glucose Levels at the End of the Study

**Figure 1:**
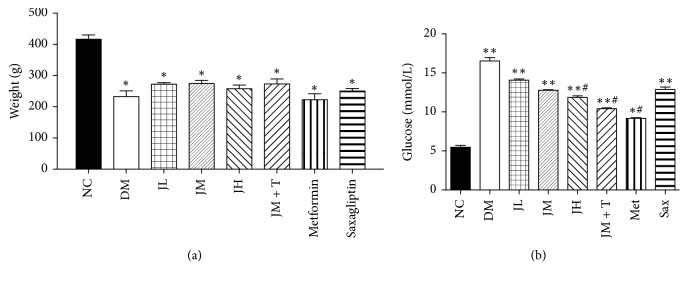
Body weight and blood glucose levels at the end of the study.

As shown in Figure 1(a), weight of rats in diabetic group were significantly lower than that in NC group (*P* < 0.05), while there were no significant differences between the drug groups and DM group. At the end of the study, blood glucose levels of rats in each group were measured. Results showed that the fasting blood glucose in diabetic group was higher than that in NC group (*P* < 0.05), which was significantly decreased in JH, JM + T, and Met group compared to DM group (*P* < 0.05) (Figure 1(b)).

### 3.2. Changes of HPT Axis Related Hormones and Receptors

**Figure 2:**
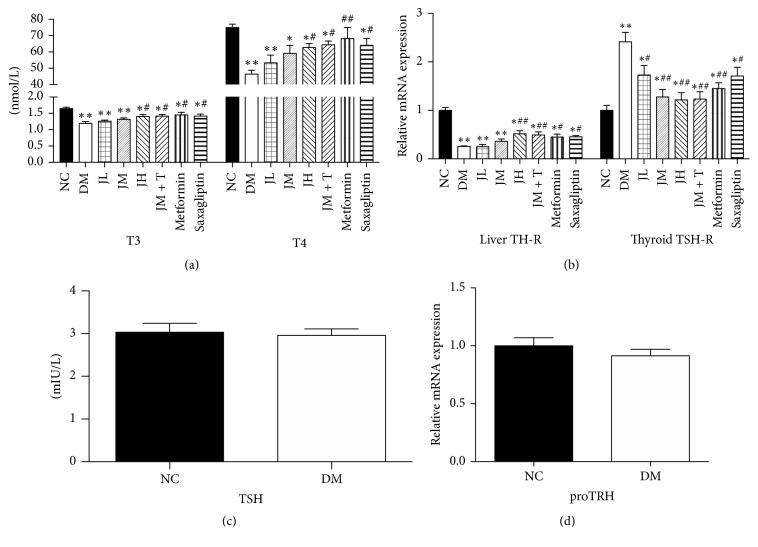
Changes of HPT axis related hormones and receptors.

The concentration of T3 and T4 in diabetic group decreased significantly (*P* < 0.01) compared with that in NC group (Figure 2(a)). There was no statistical difference between NC group and diabetic group regarding TSH concentration and level of proTRH mRNA in hypothalamus (Figures 2(b) and 2(c)). Compared with that in DM group, T3 and T4 concentration in JH, JM + T, Met, and Sax group increased significantly (*P* < 0.05, Figure 2(a)).

Compared with that in NC group, the expression level of liver TR mRNA in DM rats was significantly decreased (*P* < 0.01), while the expression level of thyroid TSHR mRNA was significantly increased (*P* < 0.01) (Figure 2(d)). Compared with that in DM group, the expression of liver TR mRNA in JH, JM + T, Met, and Sax group were significantly increased (*P* < 0.05). Compared with DM group, the expression levels of thyroid TSHR mRNA levels in JM, JH, JM + T, Met, JL, and Sax group were significantly increased (*P* < 0.05) (Figure 2(d)).

### 3.3. Levels of NIS mRNA and Protein in Thyroid Tissue

**Figure 3:**
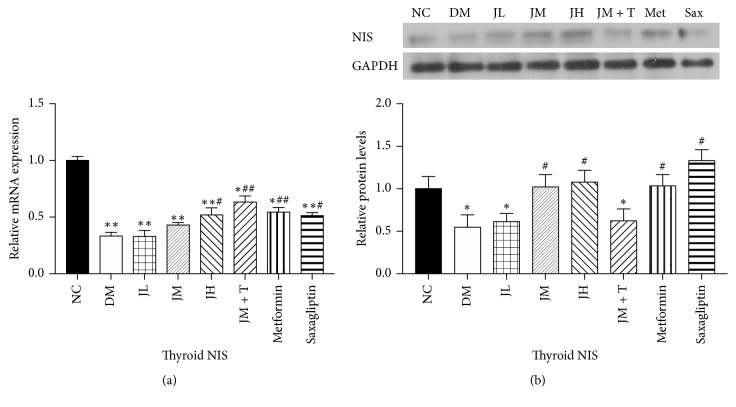
Levels of NIS mRNA and protein in thyroid tissue.

The expression of thyroid NIS mRNA in NC (*P* < 0.01), JM + T, Met (*P* < 0.01), JH, and Sax group (*P* < 0.05) was significantly increased compared with DM group, while there was no significant difference between JL, JM, and DM group (Figure 3). Levels of NIS protein in thyroid tissue were similar to those of NIS mRNA in each group.

### 3.4. Levels of Dio1 mRNA and Protein in Liver Tissue

**Figure 4:**
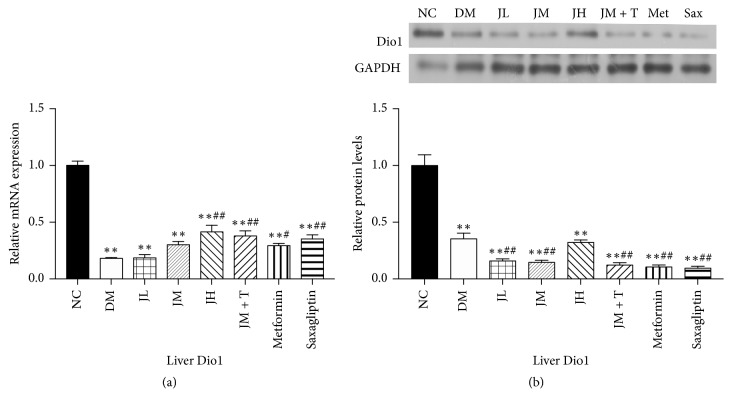
Levels of Dio1 mRNA and protein in liver tissue.

The expression of liver Dio1 mRNA in diabetic group was significantly decreased (*P* < 0.01) compared with NC group, and it was significantly decreased in JH, JM + T, Sax (*P* < 0.01), and Met group (*P* < 0.05) compared with DM group. There was no significant difference between JL, JM, and DM group (Figure 4). The levels of Dio1 protein in liver tissue were similar to those of Dio1 mRNA in each group.

### 3.5. Changes of NF-*κ*B Pathway, TNF*α*, and IL-6 Levels in Liver Tissue

**Figure 5:**
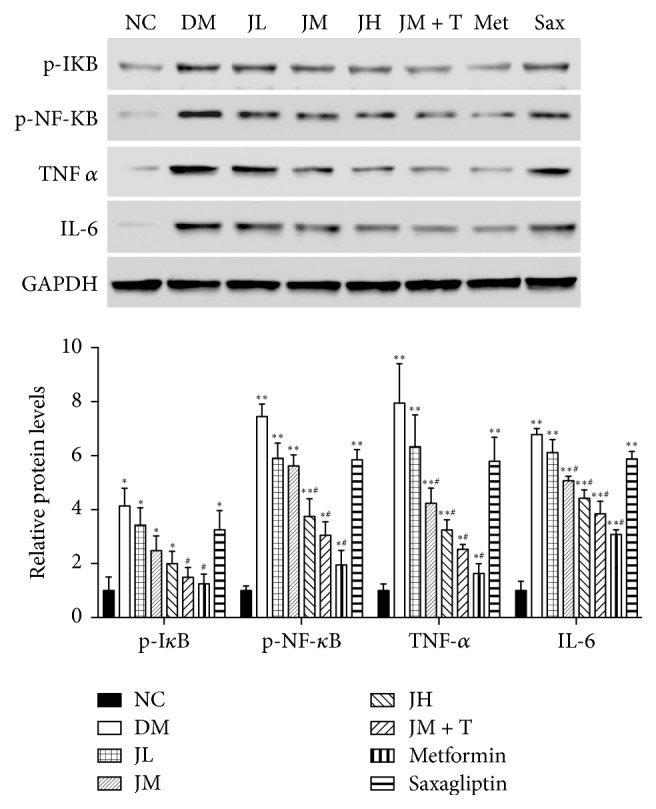
Changes of NF-*κ*B pathway, TNF*α*, and IL-6 levels in liver tissue.

Levels of liver pI*κ*B, TNF*α* (*P* < 0.05), and pNF-*κ*B, IL-6 (*P* < 0.01) in diabetic group were significantly increased compared with NC group. Levels of pI*κ*B in Met and JM + T group were significantly lower than that in DM group (*P* < 0.05). Levels of liver pNF-*κ*B, TNF*α*, and IL-6 in JH, JM + T, and Met group were significantly decreased (*P* < 0.05) compared to DM group (Figure 5).

### 3.6. H&E Staining of the Thyroids

**Figure 6:**
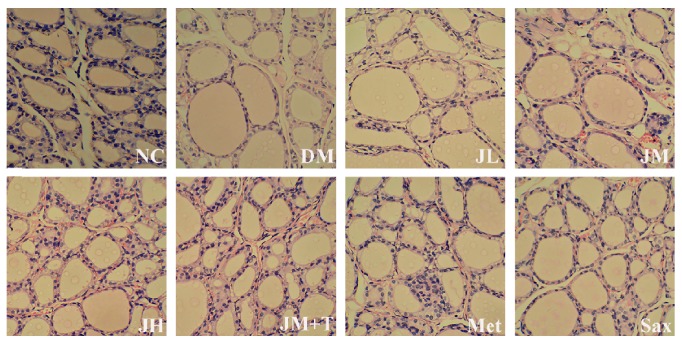
H&E staining of the thyroids.

Thyroid HE staining showed that the average thyroid follicular epithelium and follicular area decreased, and colloid area in DM rats increased, suggesting that TH synthesis, reabsorption, and secretory were impaired in DM. In contrast, the pathological change was obviously improved in JH, JM + T, and Met group (Figure 6).

## 4. Discussion

Previous reports on thyroid function in diabetic rats were inconsistent and there was no intensive exploration of HPT axis function. Our results showed significantly lower T3 and T4 levels in diabetic group than those in NC group, which was consistent with previous results [18–21]. We also detected no significant difference on the levels of TSH and pro-TRH mRNA between normal and DM group, which were in line with results of Nascimento-Saba et al. [20] and Derkach et al. [22]. However, Akbarzadeh et al. detected that TSH in diabetic rats induced by 55 mg/kg STZ was even higher than that of NC group [18], and similar result was proved in other studies using low-dose STZ (30–40 mg/kg) to induce mild diabetic model [22, 23]. The discrepancies might be related to the dose and manner of STZ administration in diabetes model. Most studies showed decreased T3 and T4 levels, as well as impaired HPT axis in DM, which was in line with nonthyroid disease syndrome (NTIS) [24]. NTIS was often observed in clinical situations such as acute sepsis, chronic inflammatory diseases, and metabolic diseases. Its pathophysiological mechanisms were poorly understood and thought to be associated with the augmented inflammatory cytokines and reactive oxygen species (ROS) [25].

We reported for the first time that the thyroid hormone receptor beta (TR*β*) decreased significantly in diabetic rats, which was different from hypothyroidism with elevated levels of TR [26], suggesting that alterations of thyroid hormone receptors could help identify hypothyroidism and NTIS in diabetics. In contrast, TSHR was significantly upregulated in diabetic rat. Thyroid stimulating hormone receptor (TSHR) is a member of the G protein-coupled receptor located on the thyroid follicular epithelial cell membrane, which mainly mediates the TSH secretion and regulates the thyroid cells function. The regulation of TSHR transcription depends on two routes. Firstly, TSH reduces the expression of thyroid nuclear transcription factor-1 (TTF-1) and inhibits the combination of TTF-1 and* TSHR*, finally reducing TSHR expression [27]. Secondly, thyroglobulin could inhibit the TTF-1 expression, leading to decreased TSHR [28]. In our study, the upregulation of TSHR mRNA in thyroid tissue might be secondary to the low TH level. The lack of insulin/IGF-1 and low level of TH in DM might result in the decreased thyroglobulin, which reduced the inhibition of* TSHR* transcription.

A recent study suggested that metformin as AMPK agonist had effects on the expression of NIS and thyroid iodine intake. TSH levels increased and T4 remained unchanged in AMPK*α* knockout rats, indicating that AMPK might improve the TH sensitivity to TSH [29]. These findings implied that the impact of anti-HPT axis dysfunction might be related to NIS, which was a glycoprotein mediating iodide transport and the synthesis of thyroid hormones. Iodine uptake was reported to be normal in some studies [30], while we detected lower NIS mRNA and protein levels in diabetic group compared with control group and similar results were found in other studies [31]. Decreased NIS transcription and expression in thyroid tissue of diabetic rats might be induced by inflammatory cytokines. Recent studies showed that ROS level in the thyroid tissue of diabetic rats increased [19] and thyroid redox state could affect binding ability of thyroid specific transcription factors such as NTF-1 with cis-acting element. Therefore, it was presumed that increased ROS levels in diabetes reduced the binding ability of NTF-1 to cis-acting elements, thereby inhibiting NIS transcription and expression.

In previous experiments with human hepatoma cell line HepG2, the binding ability of TR or its mRNA expression level decreased after treatment with inflammatory factors such as TNF*α*, IL-1, and IL-6 [32]. Moreover, in the animal model of acute inflammation induced by LPS treatment, both levels of serum TH and liver TR*α* and TR*β*1 decreased [33, 34] as reported in our results, indicating the inflammatory features of diabetes [35, 36]. Promoter of TR*β* contains three NF-*κ*B response elements, and the TNF-*α* is a common stimulator of NF-*κ*B activation. Considering the high expression of inflammatory cytokines, p-P65 and p-I*κ*B in liver tissue of diabetic rats, we speculated that inflammatory cytokines could activate NF-*κ*B pathway, which caused the decline of TR*β* mRNA. NF-*κ*B might enter the nucleus and bind to NF-*κ*B response element on TR*β* promoter to inhibit the transcription of TR*β* mRNA after I*κ*B was activated by IL-6 and TNF-*α*. Cellular study had confirmed that the decreased TR*β* expression induced by IL-1*β* was mediated by NF-*κ*B pathway [37].

As we know, only a small proportion of T3 in serum is directly secreted by the thyroid gland, and 80% of T3 is derived from the deiodination of T4 in peripheral tissues. Liver Dio1 is considered as one of the major sources of T3. So we hypothesize that the decrease of hepatic Dio1 in diabetic patients is another critical cause for the decrease of T3 in serum.

Deiodinase is reported to be positively regulated by T3 in both humans and mice studies [38, 39]. The human Dio1 promoter contains two functional T3 response elements (TREs). T3 induces transcription of deiodinase through binding into TRs-RXR dimers in TREs. The regulation of hepatic deiodinase Dio1 is mainly dependent on TR*β* because T3-induced deiodinase is significantly decreased in TR*β* knockout rats and remains normal in TR*α*1 knockout rats [40]. Therefore, decline of deiodinase Dio1 in diabetic rats might be related to the downregulation of TR*β* induced by NF-*κ*B activation.

The traditional Chinese medicine Jinlida granule is among the recommendations of the newest China Guideline for T2DM. We detected that Jinlida granules improved HPT axis function in a dose dependent manner, and the effect was even more obvious when it was combined with Tongxinluo, a traditional Chinese medicine to improve vascular function. Our results also showed that Jinlida granules could increase the expression of thyroid NIS and liver Dio. We speculated that its unique effect might be related to the anti-inflammatory effects involving NF-*κ*B pathway, but the present research was not sufficient to prove it. More studies with related inhibitors or gene knockout models were needed to unravel the exact biological mechanism.

## 5. Conclusions

We confirmed the presence of impaired HPT axis in diabetic rats, manifested as low levels of TH and hepatic TR, as well as the impaired negative feedback of TH to HPT axis. Jinlida granules could improve HPT axis function and raised the levels of TH and TR*β*. The mechanism might be associated with the anti-inflammatory effects involving NF-*κ*B pathway, while it was to be unraveled by further study. The present study suggested the potential benefit of Jinlida granules for patients with HPT axis dysfunction and DM, which was to be verified by more experimental and clinical studies.

## Figures and Tables

**Table 1:** The formula and component of Jinlida granules.

Material	Plant family	Species	Combination Principle	Origin	Medicinal Parts	Concocted
Ginseng Radix et Rhizoma	Araliaceae	Panax ginseng C.A. Mey	King	Jilin	Roots and Rhizomes	Drying
Polygonati Rhizoma	Asparagaceae	Polygonatum kingianum Coll. et Hemsl	Minister	Jilin	Rhizomes	Drying
Atractylodis Rhizoma	Compositae	Atractylodes lancea (Thunb.) DC	Minister	Jiangsu	Rhizomes	Drying
Sophorae Flavescentis Radix	Leguminosae	Sophora flavescens Ait	Minister	Neimenggu	Roots	Drying
Ophiopogonis Radix	Asparagaceae	Ophiopogon japonicas (L.f) Ker-Gawl	Assistant	Zhejiang	Tubers	Drying
Rehmanniae Radix	Plantaginaceae	Rehmanniag lutinosa Libosch	Assistant	Henan	Tubers	Drying
Polygoni multiflori Radix Praeparata	Polygonaceae	Polygonum multiflorum Thunb	Assistant	Guangdong	Tubers	Boil, Steam, Drying
Corni Fructus	Cornaceae	Cornus officinalis Sieb. et Zucc	Assistant	Henan	Flesh	Drying
Poria	Polyporaceae	Poriacocos (Schw.) Wolf	Assistant	Anhui	Sclerotia	Drying
Eupatorii Herba	Compositae	Eupatorium fortune Turcz	Assistant	Jiangsu	Aboveground parts	Drying
Coptidis Rhizoma	Ranunculaceae	Coptis chinensis Franch.	Assistant	Sichuan	Rhizomes	Drying
Anemarrhenae Rhizoma	Asparagaceae	Anemarrhena asphodgfoides Bge	Assistant	Hebei	Rhizomes	Drying
Epimedii Folium	Berberidaceae	Epimedium brevicornu Maxim	Assistant	Guizhou	Leaves	Drying
Salviae Miltiorrhizae Radix et Rhizoma	Lamiaceae	Salvia miltiorrhiza Bge.	Assistant	Jiangsu	Roots and Rhizomes	Drying
Lycii Cortex	Solanaceae	Lycium chinense Mill.	Assistant	Hebei	Velamina	Drying
Puerariae Thomsonii Radix	Leguminosae	Pueraria lobata (Willd.) Ohwi	Ambassador	Anhui	Roots	Drying
Litchi Semen	Sapindaceae	Litchi chinensis Sonn.	Ambassador	Shangdong	Seeds	Drying

**Table 2:** The composition and dosage of rats in each group.

Group	Ingredients and Dosage (Powder Weight/Rat Body Weight)
JL group	0.75 g/kg Jinlida Granules
JM group	1.5 g/kg Jinlida Granules
JH group	3.0 g/kg Jinlida Granules
JM + T group	1.5 g/kg Jinlida Granules + 0.4 g/kg Tongxinluo
Met group	50 mg/kg metformin
Sax group	1 mg/kg Saxagliptin
DM group	An equal volume of 0.5% Na-CMC
NC group	An equal volume of 0.5% Na-CMC

**Table 3:** Oligonucleotide primers used for reverse transcription-quantitative polymerase chain reaction.

Gene	Forward	Reverse
r TR-*β*	5′-TTCCAGCACCCTTACTCTC-3′	5′-GCCTCAAACCAGTCAAGTC-3′
r TSHR	5′-CTACAACCACGCCATTGAC-3′	5′-CGAGAAGGAAGCAGGAAAC-3′
r ProTRH	5′-TCTGCAGAGTCTCCACTTC GCAGACTCCAG-3′	5′-GGTGACATCAGACTCCATC CAGGGGAAGGA-3′
r Dio1	5′-GGAAGACAGGGCTGAGTATGG-3′	5′-GCTGCCGAAGTTCAACACC-3′
r NIS	5′-GAGCCACCAACGCTTCCAAC-3′	5′-AGGTCCCACCACAGCAATCC-3′
r GAPDH	5′-GTCGGTGTGAACGGATTTG-3′	5′-TCCCATTCTCAGCCTTGAC-3′
